# Crystal structure of 3-methyl-5-tri­methyl­silyl-1*H*-pyrazole

**DOI:** 10.1107/S2056989015008567

**Published:** 2015-05-13

**Authors:** Gregory M. Ferrence, Joshua L. Kocher

**Affiliations:** a CB 4160, Department of Chemistry, Illinois State University, Normal, IL 61790, USA

**Keywords:** crystal structure, pyrazole, tertramer, hydrogen bonding

## Abstract

The title compound, C_7_H_14_N_2_Si, crystallizes in a tetra­gonal space group and exists as an N—H⋯N hydrogen-bonded tetra­mer, formed around the crystallographic fourfold rotoinversion axis. The mol­ecular identity is clearly the 5-tri­methyl­silyl-3-methyl-1*H*-pyrazole tautomer and the structure is isomorphous with that of 5-*tert*-butyl-3-methyl-1*H*-pyrazole [Foces-Foces & Trofimenko (2001 ▸). *Acta Cryst.* E**57**, o32–o34].

## Related literature   

For synthetic preparation of the title compound, see: Aoyama *et al.* (1984 ▸). For isomorphous 5-*tert*-butyl-3-methyl-1*H*-pyrazole, see: Foces-Foces & Trofimenko (2001 ▸). For a general introduction to polypyrazolylborate chemistry, see: Trofimenko (1999 ▸). For related structures, see the Cambridge Structural Database: Groom & Allen (2014 ▸).
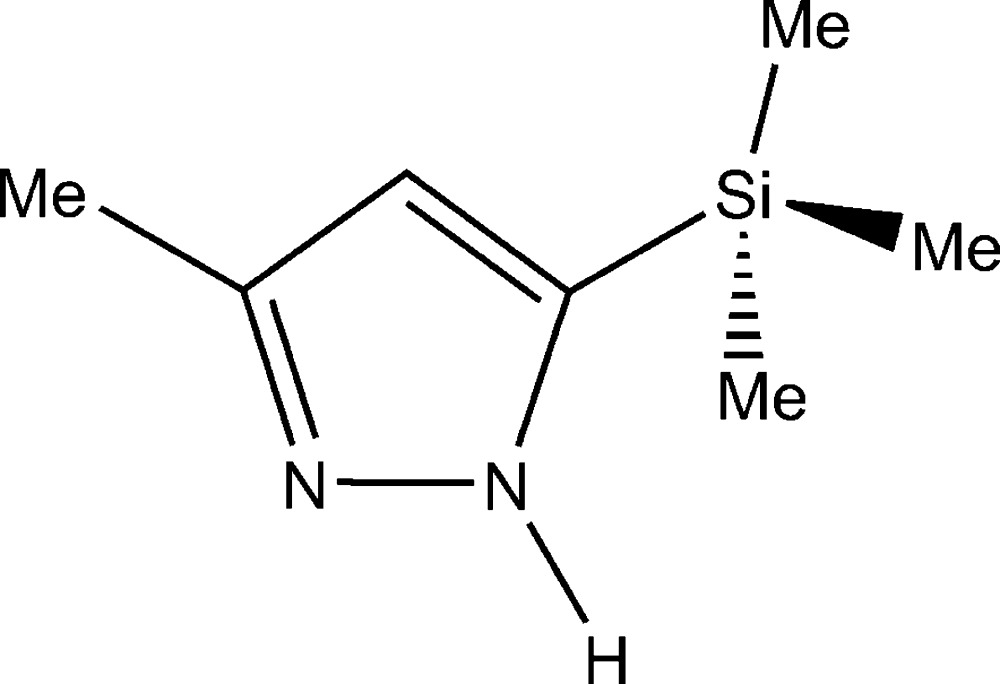


## Experimental   

### Crystal data  

C_7_H_14_N_2_Si
*M* *_r_* = 154.29
Tetragonal, 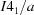

*a* = 19.221 (3) Å
*c* = 10.5812 (18) Å
*V* = 3909.4 (15) Å^3^
*Z* = 16
Mo *K*α radiation
μ = 0.18 mm^−1^
*T* = 100 K
0.3 × 0.22 × 0.09 mm

### Data collection  

Bruker SMART APEX CCD diffractometer
Absorption correction: multi-scan (*APEX*; Bruker, 2008 ▸) *T*_min_ = 0.500, *T*_max_ = 0.746
14542 measured reflections
2240 independent reflections
1811 reflections with *I* > 2σ(*I*)
*R*_int_ = 0.046

### Refinement  

*R*[*F* ^2^ > 2σ(*F* ^2^)] = 0.046
*wR*(*F* ^2^) = 0.121
*S* = 1.11
2240 reflections
100 parameters
H atoms treated by a mixture of independent and constrained refinement
Δρ_max_ = 0.38 e Å^−3^
Δρ_min_ = −0.28 e Å^−3^

### 

Data collection: *APEX2* (Bruker, 2008 ▸); cell refinement: *APEX2* and *SAINT* (Bruker, 2008 ▸); data reduction: *SAINT*; program(s) used to solve structure: *SUPERFLIP* (Palatinus & Chapuis, 2007 ▸); program(s) used to refine structure: *SHELXL2014* (Sheldrick, 2015 ▸); molecular graphics: *ORTEP-3 for Windows* (Farrugia, 2012 ▸) and *Mercury* (Macrae *et al.*, 2008 ▸); software used to prepare material for publication: *WinGX* (Farrugia, 2012 ▸) and *publCIF* (Westrip, 2010 ▸).

## Supplementary Material

Crystal structure: contains datablock(s) global, I. DOI: 10.1107/S2056989015008567/zl2621sup1.cif

Structure factors: contains datablock(s) I. DOI: 10.1107/S2056989015008567/zl2621Isup2.hkl

Supporting information file. DOI: 10.1107/S2056989015008567/zl2621Isup3.cml

**Fig. 1.**
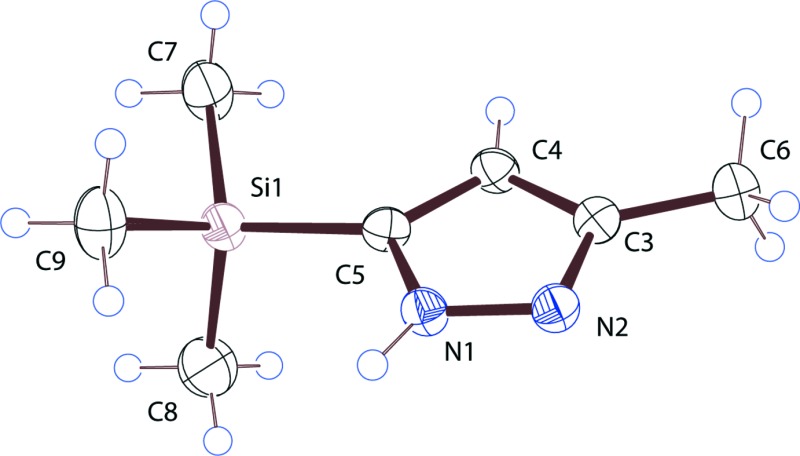
ORTEP-3 view of the title compound. Displacement ellipsoids are drawn at the 50% probability level. Only the larger part of the disordered H-atoms attached to C6 are shown.

ORTEP-3 . DOI: 10.1107/S2056989015008567/zl2621fig1.tif
*ORTEP-3* view of the title compound. Displacement ellipsoids are drawn at the 50% probability level. Only the larger part of the disordered H-atoms attached to C6 are shown.

**Fig. 2.**
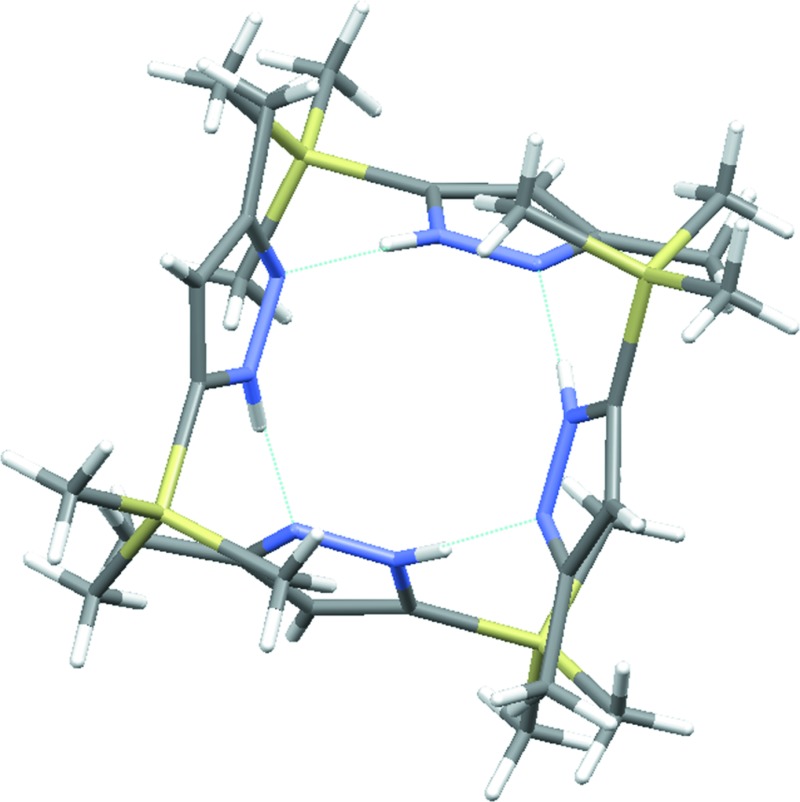
Mercury rendering of the R44(12) H-bonding motif view of the title compound.

. DOI: 10.1107/S2056989015008567/zl2621fig2.tif
Mercury rendering of the 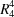
(12) H-bonding motif view of the title compound.

CCDC reference: 1062812

Additional supporting information:  crystallographic information; 3D view; checkCIF report

## Figures and Tables

**Table 1:** Hydrogen-bond geometry (, )

*D*H*A*	*D*H	H*A*	*D* *A*	*D*H*A*
N1H1N2^i^	0.87(3)	2.03(3)	2.895(2)	171(2)

Symmetry code: (i) 
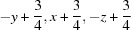
.

## supplementary crystallographic information

### S1. Chemical context

Pyrazoles, particularly 3,5-disubstituted pyrazoles have found widespread use in the preparation of tris­pyrazolylborate salts. The tris­pyrazolylborates make excellent supporting ligands for stabilization of a wide range of metal-organic coordination compounds. Most often functioning as tridentate ligands, this class of molecules is often referred to as the scorpionates (Trofimenko, 1999). We have studied a variety of tris­(3-*tert*-butyl-5-methyl)­pyrazolylborate supported metal complexes and have found the 27 proton *tert*-butyl resonance in the ^1^H NMR to often obscure important resonances from other aliphatic fragments. Replacing the *tert*-butyl group with a tri­methyl­silyl group would clear the standard aliphatic region of the NMR spectrum. During the course of our studies, we obtained X-ray quality crystals of the title compound which is reported herein.

### S2. Structural commentary

The title compound (Fig. 1) is isomorphous with known 5-*tert*-butyl-3-methyl-1*H*-pyrazole (Foces-Foces & Trofimenko, 2001). Apart from substitution of the tertiary carbon atom in 5-*tert*-butyl-3-methyl-1*H*-pyrazole with a silicon atom in the title compound, the structural features are nearly indistinguishable. At 100 K, the title structure appeared to have well ordered tri­methyl­silyl groups, while the 240 K structure of 5-*tert*-butyl-3-methyl-1*H*-pyrazole displayed rotational disorder of the *tert*-butyl group.

### S3. Supra­molecular features

Like 5-*tert*-butyl-3-methyl-1*H*-pyrazole, the title compound's packing includes an R_4_^4^(12) hydrogen-bonding motif (Fig. 2).

### S4. Database survey

Pyrazoles are ubiquitous, particularly as building blocks for pyrazolylborate metal complexes. Restricting a Cambridge Structural Database (Version 5.36, last update February 2015; Groom & Allen, 2014) search to include only those pyrazoles comprised of C, H, N, P, O, S, F, Cl, Br, I and Si returned nearly 900 entries which included over 750 different pyrazole compounds. Of these, 25 structures are in tetra­gonal space groups. Nine of these structures display the same R_4_^4^(12) hydrogen-bonding motif as the title compound. These include CSD refcode entries: AFAWEK, FAQTIA, GIRNEA, QAMQEA, QOFWUD, RIWDUX, TUHNEQ, UXOVAF, YESWUP. In all of these structures, including the title compound, a crystallographic 4-fold rotoinversion axis is at the center of the H-bonding motif.

### S5. Synthesis and crystallization

Synthesis of the title compound was accomplished following a literature procedure involving reaction of tri­methyl­silyl­diazo­methane with n-butyl­lithium, followed by reaction with the α, β-unsaturated methacrylo­nitrile (Aoyama *et al.*, 1984). X-ray quality crystals were obtained by slow evaporation of a chloro­form solution of the title compound.

### S6. Refinement

The pyrazole-H atom was located in a difference Fourier map and refined freely. All other H atoms were initially located in a difference Fourier map, but were included in the final refinement using the standard geometrically idealized positions and refined using the riding-model approximation (C–H = 0.95 and 0.98 Å for Ar–H and CH_3_; *U*_iso_(H) = 1.2*U*_eq_(C) for the aromatic H atom and *U*_iso_(H) = 1.5*U*_eq_(C)) for methyl groups. The H atoms of the C6-methyl group were refined as orientationally disordered using the AFIX 127 command in SHELX2014 (Sheldrick, 2015), with refined occupancies of 0.58 (3) and 0.42 (3) for the two moieties, respectively.

Crystal data, data collection and structure refinement details are summarized in Table 1.

### Crystal data

**Table:**

C_7_H_14_N_2_Si	*D*_x_ = 1.049 Mg m^−^^3^
*M**_r_* = 154.29	Mo *K*α radiation, λ = 0.71073 Å
Tetragonal, *I*4_1_/*a*	Cell parameters from 6620 reflections
Hall symbol: -I 4ad	θ = 2.2–31.0°
*a* = 19.221 (3) Å	µ = 0.18 mm^−^^1^
*c* = 10.5812 (18) Å	*T* = 100 K
*V* = 3909.4 (15) Å^3^	Plate, colourless
*Z* = 16	0.3 × 0.22 × 0.09 mm
*F*(000) = 1344	

### Data collection

**Table:**

Bruker SMART APEX CCD diffractometer	2240 independent reflections
Radiation source: sealed tube	1811 reflections with *I* > 2σ(*I*)
Graphite monochromator	*R*_int_ = 0.046
ω scans	θ_max_ = 27.5°, θ_min_ = 2.1°
Absorption correction: multi-scan (*APEX*; Bruker, 2008)	*h* = −24→24
*T*_min_ = 0.500, *T*_max_ = 0.746	*k* = −24→24
14542 measured reflections	*l* = −13→13

### Refinement

**Table:**

Refinement on *F*^2^	Primary atom site location: iterative
Least-squares matrix: full	Secondary atom site location: iterative
*R*[*F*^2^ > 2σ(*F*^2^)] = 0.046	Hydrogen site location: mixed
*wR*(*F*^2^) = 0.121	H atoms treated by a mixture of independent and constrained refinement
*S* = 1.11	*w* = 1/[σ^2^(*F*_o_^2^) + (0.0425*P*)^2^ + 6.7007*P*] where *P* = (*F*_o_^2^ + 2*F*_c_^2^)/3
2240 reflections	(Δ/σ)_max_ < 0.001
100 parameters	Δρ_max_ = 0.38 e Å^−^^3^
0 restraints	Δρ_min_ = −0.28 e Å^−^^3^
0 constraints	

### Special details

**Table:**

Geometry. All esds (except the esd in the dihedral angle between two l.s. planes) are estimated using the full covariance matrix. The cell esds are taken into account individually in the estimation of esds in distances, angles and torsion angles; correlations between esds in cell parameters are only used when they are defined by crystal symmetry. An approximate (isotropic) treatment of cell esds is used for estimating esds involving l.s. planes.

### Fractional atomic coordinates and isotropic or equivalent isotropic displacement parameters (Å^2^)

**Table:**

	*x*	*y*	*z*	*U*_iso_*/*U*_eq_	Occ. (<1)
Si1	0.15752 (3)	0.66265 (3)	0.08122 (5)	0.02652 (17)	
N2	−0.01542 (8)	0.63160 (8)	0.29098 (15)	0.0252 (3)	
N1	0.04990 (8)	0.65356 (8)	0.26486 (15)	0.0243 (3)	
C3	−0.03518 (10)	0.59551 (10)	0.18886 (18)	0.0242 (4)	
C4	0.01831 (10)	0.59437 (10)	0.09907 (17)	0.0252 (4)	
H4	0.0174	0.5719	0.0192	0.03*	
C5	0.07285 (10)	0.63252 (9)	0.14950 (17)	0.0233 (4)	
C7	0.17863 (12)	0.60908 (13)	−0.0598 (2)	0.0418 (6)	
H7A	0.2214	0.6265	−0.0989	0.063*	
H7B	0.1403	0.6119	−0.1207	0.063*	
H7C	0.1853	0.5605	−0.0341	0.063*	
C8	0.14560 (14)	0.75508 (12)	0.0363 (3)	0.0478 (6)	
H8A	0.1889	0.773	−0.0003	0.072*	
H8B	0.1336	0.7823	0.1115	0.072*	
H8C	0.1081	0.7588	−0.026	0.072*	
C6	−0.10613 (10)	0.56396 (11)	0.1820 (2)	0.0318 (5)	
H6A	−0.1042	0.521	0.1322	0.048*	0.58 (3)
H6B	−0.1382	0.5968	0.1416	0.048*	0.58 (3)
H6C	−0.1226	0.5534	0.2675	0.048*	0.58 (3)
H6D	−0.1391	0.5931	0.2287	0.048*	0.42 (3)
H6E	−0.1051	0.5173	0.2193	0.048*	0.42 (3)
H6F	−0.1207	0.5607	0.0934	0.048*	0.42 (3)
C9	0.22749 (12)	0.65443 (14)	0.2008 (2)	0.0422 (6)	
H9A	0.2322	0.6056	0.2259	0.063*	
H9B	0.2158	0.6826	0.2751	0.063*	
H9C	0.2715	0.6708	0.1648	0.063*	
H1	0.0738 (13)	0.6790 (13)	0.317 (2)	0.039 (7)*	

### Atomic displacement parameters (Å^2^)

**Table:**

	*U*^11^	*U*^22^	*U*^33^	*U*^12^	*U*^13^	*U*^23^
Si1	0.0269 (3)	0.0312 (3)	0.0214 (3)	−0.0041 (2)	0.0032 (2)	−0.0034 (2)
N2	0.0265 (8)	0.0282 (8)	0.0210 (8)	−0.0010 (6)	0.0025 (6)	−0.0018 (6)
N1	0.0265 (8)	0.0281 (8)	0.0182 (8)	−0.0038 (6)	0.0003 (6)	−0.0035 (6)
C3	0.0248 (9)	0.0251 (9)	0.0226 (9)	0.0008 (7)	−0.0015 (7)	0.0013 (7)
C4	0.0292 (9)	0.0265 (9)	0.0198 (9)	−0.0019 (7)	0.0000 (7)	−0.0043 (7)
C5	0.0278 (9)	0.0243 (9)	0.0178 (9)	0.0003 (7)	0.0013 (7)	−0.0007 (7)
C7	0.0378 (12)	0.0550 (14)	0.0327 (12)	−0.0121 (11)	0.0106 (9)	−0.0153 (10)
C8	0.0482 (14)	0.0380 (12)	0.0572 (16)	−0.0057 (10)	0.0120 (12)	0.0087 (11)
C6	0.0280 (10)	0.0350 (11)	0.0325 (11)	−0.0056 (8)	−0.0009 (8)	0.0010 (9)
C9	0.0326 (11)	0.0604 (15)	0.0336 (12)	−0.0079 (10)	−0.0020 (9)	0.0007 (11)

### Geometric parameters (Å, º)

**Table:**

Si1—C8	1.853 (2)	C7—H7C	0.98
Si1—C9	1.854 (2)	C8—H8A	0.98
Si1—C7	1.858 (2)	C8—H8B	0.98
Si1—C5	1.8725 (19)	C8—H8C	0.98
N2—C3	1.339 (2)	C6—H6A	0.98
N2—N1	1.353 (2)	C6—H6B	0.98
N1—C5	1.359 (2)	C6—H6C	0.98
N1—H1	0.87 (3)	C6—H6D	0.98
C3—C4	1.400 (3)	C6—H6E	0.98
C3—C6	1.494 (3)	C6—H6F	0.98
C4—C5	1.386 (3)	C9—H9A	0.98
C4—H4	0.95	C9—H9B	0.98
C7—H7A	0.98	C9—H9C	0.98
C7—H7B	0.98		
			
C8—Si1—C9	110.27 (12)	H8A—C8—H8C	109.5
C8—Si1—C7	110.65 (13)	H8B—C8—H8C	109.5
C9—Si1—C7	110.04 (12)	C3—C6—H6A	109.5
C8—Si1—C5	106.74 (10)	C3—C6—H6B	109.5
C9—Si1—C5	109.92 (10)	H6A—C6—H6B	109.5
C7—Si1—C5	109.16 (9)	C3—C6—H6C	109.5
C3—N2—N1	105.07 (15)	H6A—C6—H6C	109.5
N2—N1—C5	113.06 (15)	H6B—C6—H6C	109.5
N2—N1—H1	122.3 (16)	C3—C6—H6D	109.5
C5—N1—H1	124.6 (16)	H6A—C6—H6D	141.1
N2—C3—C4	110.33 (16)	H6B—C6—H6D	56.3
N2—C3—C6	120.56 (17)	H6C—C6—H6D	56.3
C4—C3—C6	129.10 (18)	C3—C6—H6E	109.5
C5—C4—C3	106.61 (16)	H6A—C6—H6E	56.3
C5—C4—H4	126.7	H6B—C6—H6E	141.1
C3—C4—H4	126.7	H6C—C6—H6E	56.3
N1—C5—C4	104.93 (16)	H6D—C6—H6E	109.5
N1—C5—Si1	122.45 (14)	C3—C6—H6F	109.5
C4—C5—Si1	132.25 (14)	H6A—C6—H6F	56.3
Si1—C7—H7A	109.5	H6B—C6—H6F	56.3
Si1—C7—H7B	109.5	H6C—C6—H6F	141.1
H7A—C7—H7B	109.5	H6D—C6—H6F	109.5
Si1—C7—H7C	109.5	H6E—C6—H6F	109.5
H7A—C7—H7C	109.5	Si1—C9—H9A	109.5
H7B—C7—H7C	109.5	Si1—C9—H9B	109.5
Si1—C8—H8A	109.5	H9A—C9—H9B	109.5
Si1—C8—H8B	109.5	Si1—C9—H9C	109.5
H8A—C8—H8B	109.5	H9A—C9—H9C	109.5
Si1—C8—H8C	109.5	H9B—C9—H9C	109.5
			
C3—N2—N1—C5	−0.2 (2)	C3—C4—C5—Si1	−172.39 (15)
N1—N2—C3—C4	0.6 (2)	C8—Si1—C5—N1	−72.00 (19)
N1—N2—C3—C6	−178.76 (17)	C9—Si1—C5—N1	47.58 (19)
N2—C3—C4—C5	−0.7 (2)	C7—Si1—C5—N1	168.37 (16)
C6—C3—C4—C5	178.57 (19)	C8—Si1—C5—C4	99.9 (2)
N2—N1—C5—C4	−0.2 (2)	C9—Si1—C5—C4	−140.6 (2)
N2—N1—C5—Si1	173.60 (13)	C7—Si1—C5—C4	−19.8 (2)
C3—C4—C5—N1	0.5 (2)		

### Hydrogen-bond geometry (Å, º)

**Table:**

*D*—H···*A*	*D*—H	H···*A*	*D*···*A*	*D*—H···*A*
N1—H1···N2^i^	0.87 (3)	2.03 (3)	2.895 (2)	171 (2)

Symmetry code: (i) −*y*+3/4, *x*+3/4, −*z*+3/4.
