# Supplementary figures and images for: Crystal structure of 3-methyl-5-tri­methyl­silyl-1H-pyrazole
Source: Acta Crystallogr E Crystallogr Commun. 2015 May 13;71(Pt 6):o397. doi: 10.1107/S2056989015008567 (PMC4459369; doi:10.1107/S2056989015008567)

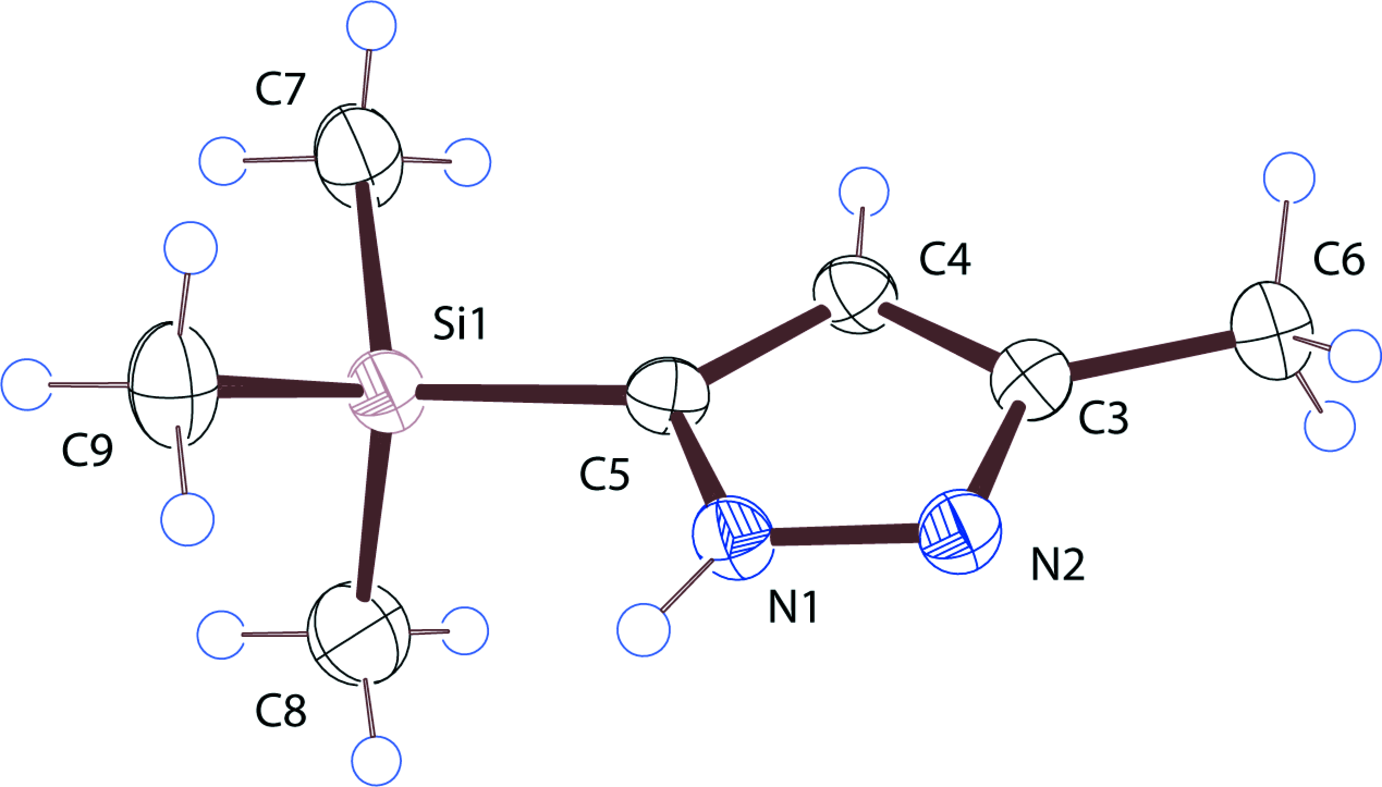

Supplement: Supplementary file 4 [file e-71-0o397-fig1.tif]

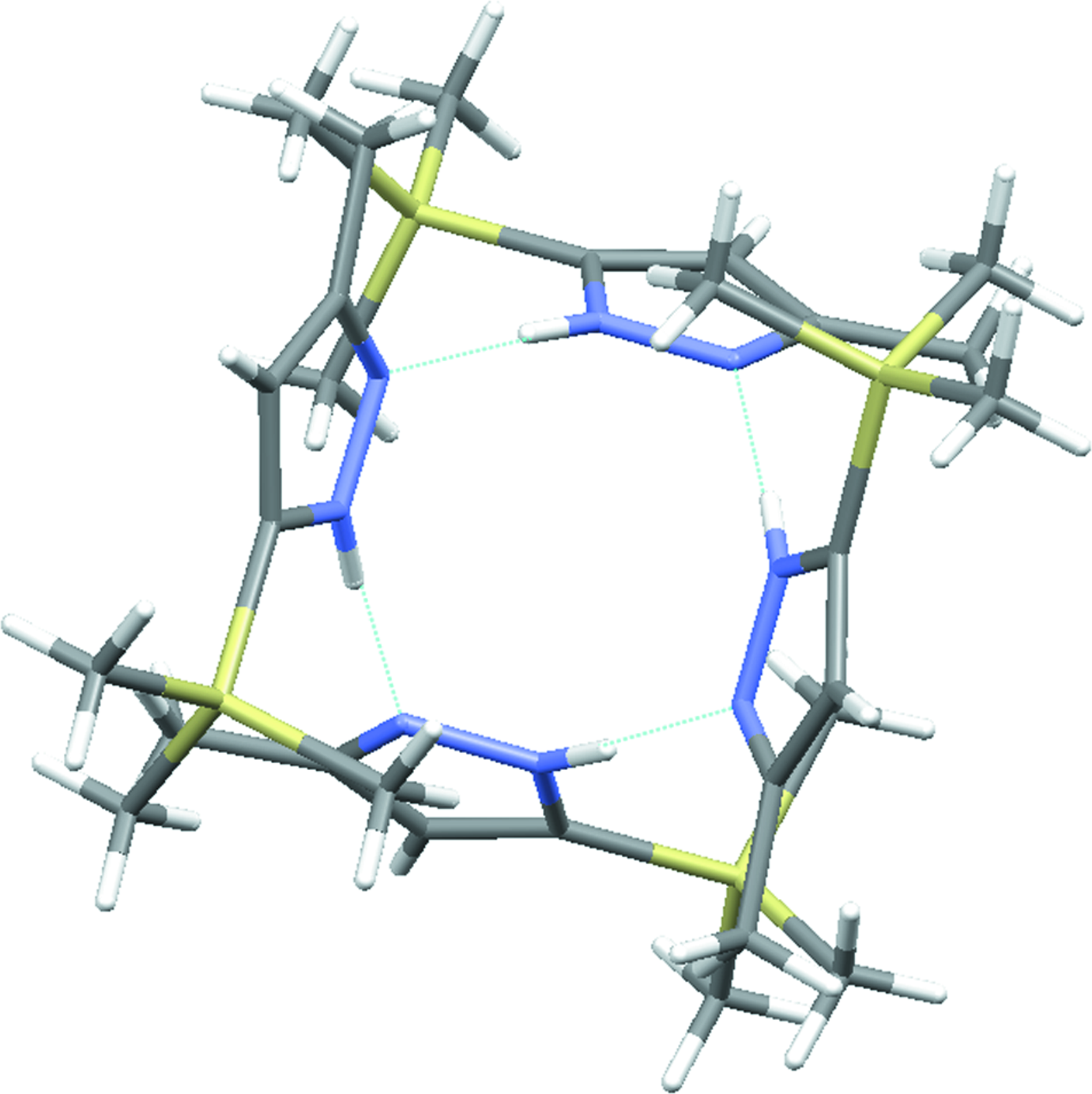

Supplement: Supplementary file 5 [file e-71-0o397-fig2.tif]
